# Computerized texture analysis of atypical immature myeloid precursors in patients with myelodysplastic syndromes: an entity between blasts and promyelocytes

**DOI:** 10.1186/1746-1596-6-93

**Published:** 2011-09-29

**Authors:** Joyce R Vido, Randall L Adam, Irene GH Lorand-Metze, Konradin Metze

**Affiliations:** 1 Department of Internal Medicine, Faculty of Medical Sciences, State University of Campinas, Rua Tessalia Vieira de Camargo 126, 13083-887, Campinas, Brazil; 2 Institute of Computing, State University of Campinas, Av. Albert Einstein 1251, 13083-852, Campinas, Brazil; 3 Department of Pathology, Faculty of Medical Sciences, State University of Campinas, Rua Tessalia Vieira de Camargo 126, 13083-887, Campinas, Brazil

**Keywords:** myelodysplastic syndromes, bone marrow, nuclear texture, cell atypias, karyometry, morphometry, fractal, chromatin, co-occurrence matrix, computerized pathology

## Abstract

**Background:**

Bone marrow (BM) blast count is an essential parameter for classification and prognosis of myelodysplastic syndromes (MDS). However, a high degree of cell atypias in bone marrow hemopoietic cells may be found in this group of clonal disorders, making it difficult to quantify precisely myeloblasts, and to distinguish them from promyelocytes and atypical immature myeloid precursors. Our aim was to investigate whether computerized image analysis of routine cytology would help to characterize these cells.

**Methods:**

In May-Grünwald-Giemsa stained BM smears of 30 newly diagnosed MDS patients and 19 cases of normal BM, nuclei of blasts and promyelocytes were digitalized and interactively segmented. The morphological classification of the cells was done by consensus of two observers. Immature granulocytic precursors, which could not be clearly classified either as blasts or promyelocytes, were called "atypic myeloid precursors". Nuclear morphometry and texture features derived from the co-occurrence matrix and fractal dimension (FD) were calculated.

**Results:**

In normal BM, when compared to myeloblasts, nuclei of promyelocytes showed significant increase in perimeter and local texture homogeneity and a decrease in form factor, chromatin gray levels, Haralick's entropy, inertia, energy, contrast, diagonal moment, cluster prominence, the fractal dimension according to Minkowski and its goodness-of-fit. Compared to normal myeloblast nuclei, the chromatin texture of MDS myeloblasts revealed higher local homogeneity and goodness-of-fit of the FD, but lower values of entropy, contrast, diagonal moment, and fractal dimension. The same differences were found between nuclei of normal promyelocytes and those of MDS. Nuclei of atypical myeloid precursors showed intermediate characteristics between those of blasts and promyelocytes according to the quantitative features (perimeter, form factor, gray level and its standard deviation), but were similar to promyelocytes according to the texture variables inertia, energy, contrast, diagonal moment, cluster prominence, and Minkowski's fractal dimension.

**Conclusion:**

BM atypical immature myeloid precursors are difficult to be correctly classified in routine cytology. Although their cytoplasm is more similar to that of myeloblasts, computerized texture analysis indicates a nuclear chromatin remodeling more close to the promyelocyte, thus indicating an asynchronous intermediate maturation stage between blast and promyelocyte.

## Background

Myelodysplastic syndromes (MDS) are a group of hemopoietic clonal disorders characterized by peripheral blood cytopenias and a cellular bone marrow (BM) showing cell atypias that reflect abnormalities in proliferation, maturation and apoptosis of hemopoietic precursors [1-6]. According to WHO criteria [7-9] the percentage of blasts counted in BM cytology is an essential parameter for diagnosis and classification of the several types of MDS, as well as for the differential diagnosis between refractory anemia with excess of blasts (RAEB) and acute myeloid leukemia. In normal hemopoiesis, strict morphological criteria can easily be used to define each stage of cell maturation, but in MDS, immature cells presenting an asynchronous maturation may often be difficult to classify [4,6-9]. Standardized morphologic criteria have been recommended [8,9] in order to separate MDS blasts and MDS promyelocytes. Yet, there is always some degree of subjectivity, although the FAB Group [1] and the International Working Group on MDS [8,9] had defined classification criteria, which were also included in the 2008 WHO classification [7]. Moreover, the European LeukemiaNet published in the Internet a consensus-based cell library elaborated by experienced morphologists [8,9] that could be used as a guide for daily work and training. Despite of these efforts, the morphologic diagnosis continues to be a difficult task, and the morphologic diagnosis of MDS should only be done by a consensus of two expert morphologists [3,6].

In cytological bone marrow smears of MDS patients, some immature cells may not be classifiable in a satisfactory way, because they show simultaneously characteristics of blasts and promyelocytes, thus not fulfilling the criteria of either category. This problem is known to the practicing hematologist, but, surprisingly not discussed in the scientific literature.

In recent years, virtual microscopy and computerized image analysis gained increasing importance [10-14]. These techniques have been widely used in pathology and cytology for the differentiation of normal cells, benign and malignant tumors [15-17], as prognostic markers in malignancy [18-20] and in order to examine chromatin remodeling of cells in culture after incubation with carcinogens [21], hormones [22] and therapeutic agents [23,24]. Computerized image analysis has shown to be a fast and reliable way for quantitative morphologic analysis [10,18,19,25-28], and moreover, to be a possibility to detect subtle morphologic changes which cannot be recognized by conventional microscopy even by an expert.

The aim of the present study was to examine whether computerized nuclear texture analysis could help to characterize in a more objective way the blasts and promyelocytes in normal bone marrow, as well as in patients with MDS. We also wanted to examine if this technology was able to classify atypical immature myeloid cells present in bone marrow smears of these patients.

## Methods

### Patients

Routinely May-Grünwald-Giemsa-stained bone marrow (BM) slides from 19 morphologically normal bone marrows and from 30 consecutive cases of MDS were used for this analysis. Morphologically normal BM smears (control group) were obtained from the diagnostic work-up of patients with idiopathic thrombocytopenic purpura, with hypersplenism, and non-Hodgkin's lymphoma without bone marrow involvement. The diagnosis of MDS was based on the presence of sustained peripheral (PB) cytopenias, cell atypias in BM cytology and BM cytogenetics according to WHO 2008 criteria [7-9].

This project was approved by the Ethic's Comitee of our Institution (Proc 0652.0.146.000-08).

### Image analysis

**Figure 1:**
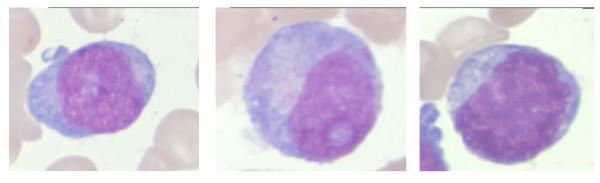
**Immature granulocytic precursors observed in the MDS cases**. A: myeloblast. B: promyelocyte. C: atypical immature granulocytic precursor. May-Grünwald-Giemsa ×1000.

At least 30 consecutive nuclei of each type of immature granulocytic precursors (myeloblasts and promyelocytes) per patient were analyzed. Cells were classified by two independent experts (JRV and ILM) according to the criteria of the European Leukemianet Project [9] (Figure 1). We considered as "atypical immature myeloid precursors" (Figure 1C) cells without cytoplasmic Golgi apparatus, but with nuclear characteristics resembling more mature cells than myeloblasts, and that did not fulfill the proposed criteria for classification [8,9].

Cell images were captured by a Leica DC 500 digital system (bmp-format; sample spacing of 0.1 μm/pixel, 1.25 numerical aperture, 100x oil immersion objective). The nuclear images were interactively segmented, converted to grayscale format with gray levels ranging between 0 and 255 (being 255 the brightest). We examined variables of geometric morphometry such as nuclear area, form factor, mean gray level, and standard deviation of gray values. We also calculated texture features derived from the co-occurrence matrix [17,27,28] and the fractal dimension (FD) according to Minkowski-Bouligand after pseudo-3D transformation [18,20], as well as its goodness of fit (R^2^) [19,20,29].

### Statistical analysis

We compared the values obtained for normal myeloblasts and promyelocytes for all nuclear morphometric and texture features using the t-test for paired values. Student's t-test was used to compare morphometric and texture features of normal and MDS blasts and of normal and MDS promyelocytes. In a third step, the differences between features of blasts, promyelocytes and atypical myeloid precursors of MDS patients were calculated by analysis of variance for repeated measures. Differences between blast and atypical myeloid precursors or between the latter and promyelocytes were calculated with the help of t-tests for paired values.

For every nuclear morphometric or texture feature we tried to find out whether the values of the atypical myeloid precursors were more similar to those of MDS-blasts (no significant difference between atypical precursors and blasts, but between atypical precursors and promyelocytes), or MDS-promyelocytes (significant difference between blasts and atypical precursors but no significant difference between atypical precursors and promyelocytes). A nuclear characteristic was considered to be "intermediate" between MDS-blasts and MDS-promyelocytes, when significant differences were found both between MDS-blasts and atypical precursors and between atypical precursors and MDS-promyelocytes. A variable was called "not defined", when the global test or both dependent t-tests were not significant (p > 0.05).

## Results

Diagnostic smears from 19 normal BM and 30 cases of MDS were examined. Median age of the patients with normal BM was 57 years (24 - 85; 9 males and 10 females). The median age of the MDS patients was 64 years (30 - 85). There were 16 males and 14 females (Table 1). According to the WHO type, most cases were RCMD (refractory cytopenia with multilineage dysplasia) and RAEB (refractory anemia with excess of blasts).

**Table 1:** Age and hematological data of the MDS cases (median)

WHO type	RA/RARS*	RCMD**	RAEB***
Number of cases	4	15	11
Age (years)	64	64	62
Hemoglobin (g/dL)	10.5	10.3	8.1
PB leukocytes (×10^3^/mm^3^)	4.7	3.6	3.1
PB platelets (×10^3^/mm^3^)	234	240	184
BM Blasts %	2.0	1.0	8.0

* RA/RARS = refractory anemia/sideroblastic anemia; ** RCMD = refractory cytopenia with multilineage dysplasia; *** RAEB = refractory anemia with excess of blasts

In normal BM, nuclei of promyelocytes showed significant changes, such as an increase of perimeter and local texture homogeneity and a decrease in form factor, chromatin gray levels, Haralick's entropy, inertia, energy, contrast, diagonal moment, cluster prominence, Minkowski's fractal dimension and its goodness-of-fit when compared with that of normal blasts (Table 2).

**Table 2:** Mean values of the variables obtained for normal granulocytic precursors

	Blasts	Promyelocytes	*P*
Area μ^2^	111.9	112.7	0.75
Perimeter	579	636	<0.0001
Form factor	0.957	0.792	<0.0001
Mean gray level	129.3	120.8	<0.0001
SD gray level*	8.6	8.1	0.04
			
Haralick's entropy	7.90	7.77	0.02
Inertia	4.17	3.82	<0.0001
Local homogeneity	0.535	0.538	0.51
Energy	7177	6430	<0.0001
Contrast	4.17	3.82	0.005
Diagonal Moment	19.1	17.7	<0.0001
Cluster prominence	3.38	2.79	0.01
			
FD** Minkowski	2.134	2.128	0.08
R^2 ^***	0.99647	0.99613	0.007

* SD = standard deviation; ** FD = fractal dimension, ***R^2 ^goodness-of-fit of the fractal dimension

Compared to normal myeloblast nuclei, chromatin texture of MDS blasts had a higher local homogeneity as well as goodness-of-fit of the fractal dimension, but a lower entropy, contrast, diagonal moment, and fractal dimension (Table 3). Nuclei of promyelocytes from MDS showed a larger nuclear area, local homogeneity and goodness-of-fit of FD, but lower inertia, entropy, energy, contrast, diagonal moment and FD than normal ones (Table 4).

**Table 3:** Mean values of the variables that differed in normal myeloblasts and those of MDS

	Normal	MDS	*p*
Haralick's entropy	7.90	7.65	0.01
Inertia	4.17	3.67	0.05
Local homogeneity	0.535	0.553	0.01
Energy	7177	6749	n.s
Contrast	4.17	3.67	0.05
Diagonal moment	19.1	17.4	0.05
FD Minkowski *	2.134	2.122	0.01
R^2^**	0.99647	0.99819	<0.005

* FD = fractal dimension. **R^2 ^goodness-of-fit of the fractal dimension

**Table 4:** Mean values of the variables that differed in normal promyelocytes and those of MDS

	Normal	MDS	*p*
Haralick's entropy	7.77	7.52	0.03
Inertia	3.82	3.28	0.01
Local homogeneity	0.538	0.558	0.01
Energy	6430	5595	0.04
Contrast	3.82	3.28	0.01
Diagonal moment	17.7	15.6	0.02
FD Minkowski *	2.128	2.118	0.03
R^2 ^**	0.99613	0.99809	0.001

* FD = fractal dimension. **R^2 ^goodness-of-fit of the fractal dimension

The values obtained for nuclei of granulocytic precursors in MDS are presented in Table 5.

**Table 5:** Mean values of the variables in the granulocytic precursors in MDS

Nuclear morphometric or texture feature	MDS-Blasts	*P *MDS Blast vs atyp	Atyp.	*p *atyp. vs. MDS Pro.	MDS Pro	*P *global	Classification of atypical myeloid precursors
Área [μ2 ]	118.4	0.95	118.5	0.37	121.0	0.60	(not defined)
Perimeter	598	0.01	628	0.004	656	0.00001	Intermediate
Form factor	0.94	<0.0001	0.85	0.0005	0.80	0.0001	Intermediate
Mean gray level	125.1	0.0009	121.1	0.0227	118.6	<0.00001	intermediate
SD* gray value	9.1	<0.0001	8.4	0.02	8.1	0.01	Intermediate
							
Haralick's entropy	7.65	0.11	7.59	0.083	7.52	0.01	(not defined)
Inertia	3.67	0.0038	3.39	0.095	3.28	0.0001	close to MDS Pro.
Local homogeneity	0.553	0.32	0.555	0.23	0.558	0.12	(not defined)
Energy	6749	0.00003	5748	0.39	5595	<0.0001	close to MDS Pro.
Contrast	3.67	0.0038	3.39	0.095	3.28	0.0001	close to MDS Pro.
Diagonal moment	17.4	0.00058	16.1	0.098	15.6	<0.0001	close to MDS Pro.
Cluster prominence (10^-5^)	3.17	0.021	2.39	0.51	2.19	0.018	close to MDS Pro.
							
FD****	2.1225	0.0015	2.1183	0.82	2.1180	0.0015	close to MDS Pro.
R2*****	0.99819	0.026	0.99807	0.65	0.99809	0.068	(not defined)

* atyp. = immature atypical myeloid precursors; **pro. = promyelocyte; *** SD = Standard deviation of gray value levels, **** FD = fractal dimension, *****R2 goodness-of-fit of the fractal dimension

Nuclei of atypical myeloid precursors showed intermediate characteristics between those of blasts and promyelocytes according to the quantitative features perimeter, form factor, gray level and its standard deviation, but were similar to promyelocytes according to the texture variables inertia, energy, contrast, diagonal moment, cluster prominence and Minkowski's fractal dimension. None of the features studied showed closeness to myeloblasts. These findings were independent of the WHO type of MDS.

## Discussion

In recent years, computerized image analysis has been widely used in histology and cytology in order to examine tissue differentiation, for tumor classification [11,12,15-17] and for the search of new prognostic variables in neoplasias [18-20]. This approach has also been used in basic research for analyzing nuclear texture changes that reflect chromatin remodeling of cells after incubation with carcinogens [21], hormones [22] and therapeutic agents [23,24].

Chromatin remodeling, which is primarily due to epigenetic events, can be found during cell differentiation or malignant transformation [20,21,23,24,26]. In normal hemopoiesis, gene expression during normal cell maturation is controlled by genetic and epigenetic changes [30]. Blast cells in acute myeloid leukemia and MDS always present epigenetic abnormalities and their DNA methylation signature is different to that of any stage of normal myeloid maturation, an observation which permits to distinguish normal and leukemic blasts [30-33].

In the present study we examined the utility of computerized chromatin texture analysis for the diagnosis of normal and atypical immature myeloid precursors in routine BM smears. In normal BM, all except three of the quantitative features examined, presented significant differences between blasts and promyelocytes. Thus, chromatin texture analysis in routine BM cytology is able to define cells in early stages of myeloid maturation.

In MDS, genetic and epigenetic alterations provoke abnormalities of proliferation, maturation, and apoptosis [5,6,32-34], which, of course, are reflected in subtle alterations of the chromatin structure. The expression of various lineage and maturation-related membrane proteins may be discordant in granulopoietic precursors [32-34] provoking also morphological atypias. All these alterations hamper the cell classification. The difficulties in classify several cases with MDS are well known. The European LeukemiaNet created a consensus-based cell library elaborated by experienced morphologists and downloaded it in the Internet [9]. Its purpose is to be a guide for daily work and training. Furthermore, it is still recommended that the morphologic diagnosis of MDS should be achieved by a consensus of two experienced morphologists [3,6]. Our study underlines these problems of classification. The cells diagnosed as atypical immature myeloid precursors in the present study did not - on light microscopic examination - reveal a Golgi apparatus, and would be therefore morphologically classified as blasts. However, the nucleus showed features more close to that of promyelocytes or nuclei of an "intermediate" state between blasts and promyelocytes.

Computerized texture analysis confirmed this subjective impression, since 4 features presented intermediate values between blasts and promyelocytes and 6 variables pointed out a similarity with nuclei of promyelocytes. Although the cytoplasm in these atypical cells still reveals characteristics of a blast, the nuclear structure is more similar to a promyelocyte, thus indicating an asynchronous maturation in MDS patients.

The existence of this intermediate maturation stage is of clinical importance. Cytologists basing their diagnosis mainly on cytoplasmic criteria will count these atypical immature cells together with blasts and thus increase the blast count. Other observers, emphasizing the similarity of the nuclear features, might count them together with promyelocytes, or count them separately, thus diminishing the blast count. This may imply in a different classification for the patient.

The BM blast count is considered very important for the classification of MDS in the revised WHO classification [7] as well as for the determination of the IPSS and WPSS scores [2,31,32]. Furthermore, the blast count is considered to be an independent prognostic feature of utmost importance in MDS [2,6,7,33,35-37]. Therefore, additional pathophysiological and molecular studies should be performed in order to investigate whether these atypical precursor cells should not be counted as blasts, as suggested by our investigation.

## Competing interests

The authors declare that they have no competing interests.

## Authors' contributions

JRV: collected all the clinical data, selected the material, classified the cells, acquired and segmented the cell images and reviewed the literature. RLA: developed the segmentation software and made all the computational analysis. ILM: provided the conception and design of the study, microscopic analysis, classification of the cells, drafting of the manuscript, and final approval. KM: participated in the study design, performed the statistical analyses and interpretation of the data, and made the critical revision of the text.

This study is part of a master thesis of JRV (Postgraduate Course in Medical Pathophysiology, FCM, University of Campinas) with ILM as principal advisor and KM as co-advisor. All authors have read and approved the manuscript.
